# Association between basal septal hypertrophy and left ventricular geometry in a community population

**DOI:** 10.1186/s12872-022-03004-x

**Published:** 2022-12-31

**Authors:** Lan Gao, Wei Ma, Min Li, Ying Yang, Litong Qi, Baowei Zhang, Chonghui Wang, Yan Zhang, Yong Huo

**Affiliations:** 1 Division of Cardiology, Department of Cardiovascular Disease, Peking University First Hospital, Dahongluochang Street, Xicheng District, Beijing, 100034 China; 2 Key Laboratory of Molecular Cardiovascular Sciences (Peking University), Ministry of Education, Beijing, China; 3 Echocardiography Core Lab, Institute of Cardiovascular Disease at Peking University First Hospital, Beijing, China

**Keywords:** Basal septal hypertrophy, Left ventricular geometry, Echocardiography

## Abstract

**Background:**

Left ventricular (LV) geometry is closely associated with cardiovascular disease; however, few studies have evaluated the relationship between basal septal hypertrophy (BSH) and LV geometry. In this study, we examined the relationship between BSH and LV geometry in a Beijing community population.

**Methods:**

The clinical and echocardiographic data of 1032 participants from a community in Beijing were analyzed. BSH was defined as a basal interventricular septal thickness ≥ 14 mm and a basal septal thickness/mid-septal thickness ≥ 1.3. On the basis of their echocardiographic characteristics, patients were described as having a normal geometry, concentric remodeling, concentric hypertrophy, or eccentric hypertrophy. Multivariable logistic regression was used to analyze the relationship between BSH, LV mass index (LVMI), and relative wall thickness (RWT).

**Results:**

The prevalence of BSH was 7.4% (95% confidence interval [CI] 5.8–9.0%). Basal and middle interventricular septal thickness, LV posterior wall thickness, and RWT were greater, while LVMI and LV end-diastolic dimension were lower in the BSH group than in the non-BSH group (*p* < 0.05). The BSH group accounted for the highest proportion of patients with concentric remodeling. A multivariable regression analysis showed that BSH increased by 3.99-times (odds ratio [OR] 3.99, 95% CI 2.05–7.78, *p* < 0.01) when RWT was > 0.42, but not when LVMI increased (OR 0.16, 95% CI 0.02–1.19, *p* = 0.07). There were no interactions between BSH and age, body mass index, sex, diabetes mellitus, coronary heart disease, stroke, and smoking in relation to an RWT > 0.42.

**Conclusion:**

BSH was independently associated with an RWT > 0.42.

## Background

The rate of basal septal hypertrophy (BSH) is approximately 10% in the general population [1]. However, the prevalence of BSH varies by definition, age group, and comorbidities. For example, a previous study reported that the rate of BSH is 18% in older individuals, while the rate of BSH in hypertensive cohorts is approximately 20% [2].

BSH is not independently associated with an adverse cardiovascular prognosis [2]. However, some studies have demonstrated that BSH is an early manifestation of hypertension. Therefore, self-measured blood pressure and ambulatory blood pressure monitoring should be performed in all patients to improve the detection of hypertension [3]. Significant BSH is associated with left ventricular (LV) outflow tract obstruction and heart failure with preserved ejection fraction [1, 4], as well as with impaired LV diastolic function [2]. Moreover, LV geometry is closely associated with cardiovascular disease, especially in patients with hypertension [5]. However, few studies have evaluated the relationship between BSH and LV geometry. Therefore, we aimed to explore the relationship between BSH and LV geometry in a Beijing community population.

## Methods

### Population

All residents who lived in the Shi Jing Shan District of Beijing and who were aged > 40 years were invited to participate. The investigation methods have been published previously [6]. Of 5593 subjects, 1069 volunteered to participate. The investigation started in 2004 and ended in 2005. Participants who underwent echocardiography were recruited, while participants who had regional wall movement abnormalities, moderate or severe aortic valve stenosis, rheumatic heart disease, or congenital heart disease were excluded. Finally, 1032 participants were included. The study was approved by the institutional review board of Peking University First Hospital, and informed consent was obtained from all participants.

### Definition of cardiovascular risk factors and disease

The methods used to measure height, weight, blood pressure, heart rate, fasting blood glucose, oral glucose tolerance, and blood lipid concentrations have been described previously [7]. Current smokers and participants with a history of smoking were defined as smokers. Hypertension was defined as a systolic blood pressure of ≥ 140 mmHg and/or a diastolic blood pressure of ≥ 90 mmHg or a history or usage of antihypertensive drugs. Diabetes mellitus was diagnosed according to each participant’s history. Participants with a fasting blood glucose concentration of ≥ 7.0 mmol/L and a 2 h glucose concentration of ≥ 11.1 mmol/L were also defined as having diabetes mellitus. BMI ≥ 28 kg/m^2^ was defined as obesity. Stroke, including intracerebral hemorrhage, cerebral infarction, and transient ischemic attack, was defined by the patient’s history. A history of myocardial infarction, percutaneous coronary intervention, and coronary artery bypass grafting were all included in coronary heart disease (CHD).

### Echocardiography

Echocardiography was performed using a 3 MHz transducer and an ultrasound system (Vivid-7; General Electric). According to previously published guidelines [8], standard images were collected and stored. One experienced clinician who was blinded to the clinical picture of the participants measured the echocardiography parameters at the central laboratory of Peking University First Hospital.

For the patients without BSH, LV end-diastolic dimension (LVEDD), LV end-systolic dimension and wall thicknesses (LVESD) including middle IVS thickness (MIVST), and LV posterior wall thickness (LVPWT) were measured at the mitral chordae level by parasternal long-axis view by 2 D method according to ASE guideline [8], basal interventricular septal thickness (BIVST) was measured simultaneously. For the patients with BSH, MIVST, LVPWT, LVEDD and LVESD were measured below the basal hypertrophy where the septal thickness was uniform, maximal BIVST thickness was measured simultaneously. LVEF was calculated by Teichholtz method. Left atrial diameter (LAD) was anteroposterior (AP) linear dimension obtained from the parasternal long-axis view in 2D image according to ASE guideline [8]. LV mass (LVM) was calculated as follows: LVM = 0.8 × 1.04 × ([PWTd + SWTd + LVIDd]^3^ − [LVIDd]^3^) + 0.6 g, where PWTd and SWTd are the posterior and middle septal wall thicknesses at LV end-diastole, respectively, and LVIDd is the LV dimension at end-diastole. LVM index (LVMI) was then calculated, as previously described [8].Relative wall thickness (RWT) was calculated using the following formula: (2 × LV PWT) ÷ LVEDD. An LVMI > 115 g/m^2^ (male) or > 95 g/m^2^ (female) was defined as increased LVMI, and RWT > 0.42 was defined as increased RWT as well. Normal geometry is defined as increased LVMI = 'NO' and increased RWT = 'NO' while concentric remodeling as increased LVMI = 'NO' and increased RWT = 'YES'. Concentric hypertrophy is defined as increased LVMI = = 'YES' and increased RWT = = 'YES' while eccentric hypertrophy as increased LVMI = 'YES' and increased RWT = 'NO' [8]

BSH was defined when all three of the following criteria were fulfilled [8]: (1) a basal IVS thickness ≥ 14 mm; (2) a basal IVS thickness/mid IVS thickness ≥ 1.3; and (3) no wall motion abnormalities or scarring in the middle septum that could result in isolated septal thickening.

### Statistical analysis

Continuous data with normal distribution are presented as mean ± standard deviation, while presented as median plus quartile when the data are abnormal distribution. Count data are presented as percentages. Student t test was used to compare between the two groups when continuous data are normal distribution. Continuous data were compared between the two groups using non-parametric test (Median Test for k samples) when the data are abnormal distribution. Categorical data were compared between the two groups using the Chi square test or the Fisher’exact test if needed.

Multivariable logistic regression was used to analyze the relationship between BSH, the increase in LVMI, and the increase in RWT, adjusting for age, sex, **obesity**, hypertension, diabetes mellitus, and heart rate concerned about univariable logistic regression analysis results and clinical significance. Subgroup analyses and interaction tests were used to examine the relationship between BSH and the increase in RWT according to age (< 60 years and ≥ 60 years), sex (male and female), BMI (< 24 kg/m^2^ and ≥ 24 kg/m^2^), diabetes mellitus (yes or no), CHD (yes or no), stroke (yes or no), and smoking status (yes or no) by multivariable logistic regression. The intraclass correlation coefficient was used to evaluate intra-observer consistency. A two-sided *p* value of < 0.05 was considered statistically significant for all tests. All analyses were performed using statistical software (Empower (R) [www.empowerstats.com]; X&Y solutions, Inc., Boston, MA, USA; R [http://www.R-project. org] v3.4.3; SPSS v13.0).

## Results

The intra-observer values for BIVST and MIVST were 0.86 (95% confidence interval [CI] 0.65–0.96, *p* < 0.01) and 0.81 (95% CI 0.52–0.93, *p* < 0.01), respectively. The general characteristics of the participants are shown in Table 1. The median age of the participants was 65 years, and 51.8% of the participants were male. The prevalence of BSH was 7.4% (95% CI 5.8–9.0%). Participants in the BSH group were older. The prevalence of diabetes mellitus and obesity were also higher in the BSH group than in the non-BSH group (*p* < 0.05).

**Table 1:** General characteristics of participants

	Total (n = 1032)	BSH (n = 76)	Non BSH (n = 956)	*p*
Age (years)	65 (56, 71)	68 (62, 72)	65 (56, 71)	0.04
Sex (male, n, %)	535 (51.8)	43 (56.6)	492 (51.5)	0.39
BMI (kg/m^2^)	25.7 (23.5, 27.9)	26.3 (23.8, 28.7)	25.8 (23.5, 27.9)	0.69
SBP (mmHg)	134 (121, 146)	138 (122, 147)	134 (121, 145)	0.44
DBP (mmHg)	80 (72, 87)	80 (74, 87)	80 (72, 87)	0.73
HR (beats/min)	77 ± 11	77 ± 11	77 ± 11	0.74
TC (mmol/l)	5.2 (4.6, 5.9)	5.2 (4.7, 5.8)	5.2 (4.6, 5.9)	0.91
TG (mmol/l)	1.8 (1.2, 2.7)	1.8 (1.3, 2.6)	1.8 (1.2, 2.7)	0.91
Smoking (n, %)	366 (35.5)	30 (39.5)	464 (48.5)	0.45
Hypertension (n, %)	824 (79.8)	64 (84.2)	760 (79.5)	0.32
Obesity (n, %)	245 (23.8%)	26 (34.2%)	219 (23.0%)	0.03
Stroke (n, %)	169 (16.4)	13 (17.1)	156 (16.3)	0.86
CHD (n, %)	130 (12.6)	8 (10.5)	122 (12.8)	0.72
Diabetes (n, %)	305 (29.6)	35 (46.1)	270 (28.2)	< 0.01

Continuous data are presented as median (IQR)

Chi square test was used and Fisher exact test when adequate

*BSH* Basal septal hypertrophy; *BMI* Body mass index; *SBP* Systolic blood pressure; *DBP* Diastolic blood pressure; *HR* Heart rate; *TC* Total cholesterol; *TG* Triglyceride; *CHD* Coronary heart disease

The echocardiographic parameters of the participants are shown in Table 2. Compared with levels in the non-BSH group, basal and middle IVS thickness, LVPW thickness, and RWT were greater, while LVMI and LVEDD were lower in the BSH group (*p* < 0.01). The BSH group accounted for the highest proportion of participants with concentric remodeling, with approximately 84.2% of participants being from the BSH group and 48.7% of participants being from the non-BSH group (*p* < 0.01).

**Table 2:** Echocardiographic parameters of participants

	Total (n = 1032)	BSH (n = 76)	Non BSH (n = 956)	*p*
BIVST (mm)	9.4 (8.8, 10.4)	14.5 (14.2, 15.2)	9.3 (8.8, 10.1)	< 0.01
MIVST (mm)	9.2 (8.7, 9.8)	9.8 (9.0, 10.3)	9.2 (8.7, 9.8)	< 0.01
LVPWT (mm)	9.2 (8.7, 9.6)	9.5 (9.0, 9.9)	9.2 (8.7, 9.6)	< 0.01
LVMI (g/m^2^)	76.0 (66.0, 86.2)	68.1 (61.4, 77.6)	76.4 (66.5, 86.9)	< 0.01
LVEDD (mm)	43.0 (40.0, 47.0)	39.0 (36.3, 42.8)	43.0 (40.0, 47.0)	< 0.01
LVEF (%)	69.0 (63.0, 75.0)	69.0 (62.0, 74.8)	69.0 (63.0, 75.0)	0.62
LAD (mm)	35.0 (32.0, 37.0)	35.0 (31.0, 37.0)	35.00 (32.0, 37.0)	0.97
RWT	0.4 (0.4, 0.5)	0.5 (0.4, 0.5)	0.4 (0.4, 0.5)	< 0.01
IVS ratio	1.0 (1.0, 1.0)	1.6 (1.4, 1.6)	1.0 (1.0, 1.0)	< 0.01
NG (n, %)	428 (41.5)	11 (14.5)	417 (43.6)	< 0.01
CR (n, %)	530 (51.4)	64 (84.2)	466 (48.7)	
CH (n, %)	26 (2.5)	0 (0)	26 (2.7)	
EH (n, %)	48 (4.7)	1 (1.3)	47 (4.9)	

Continuous data are presented as median (IQR)

Chi square test was used and Fisher exact test when adequate

*BSH* Basal septal hypertrophy; *BIVST* Basal interventricular septum thickness; *MIVST* Middle interventricular septum thickness; *LVPWT* Left ventricular posterior wall thickness; *LVMI* Left ventricular mass index; *LVEDD* Left ventricular end-diastolic dimension; *LVEF* Left ventricular ejection fraction; *LAD* Left atrial diameter; *RWT* Relative wall thickness; *IVS ratio* A basal septal thickness/mid-septal thickness; *NG* Normal geometry; *CR* Concentric remodeling; *CH* Concentric hypertrophy; *EH* Eccentric hypertrophy

**Fig. 1:**
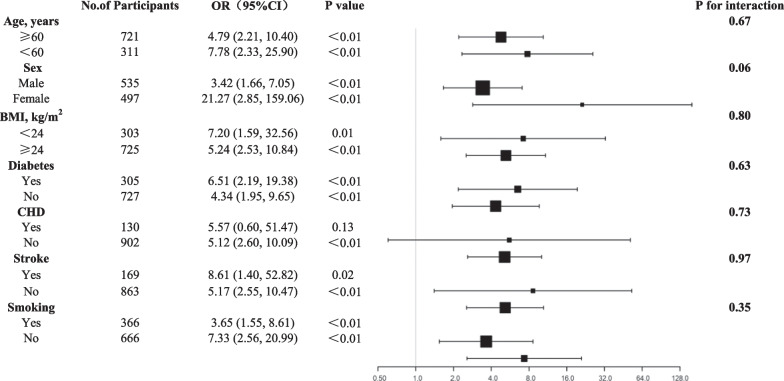
Subgroup analysis of the relationship between BSH and RWT > 0.42. *BMI* Body mass index; *BSH* Basal septal hypertrophy; *CHD* Coronary heart disease; *RWT* Relative wall thickness. Adjusted for age, sex, body mass index, hypertension, diabetes mellitus, CHD, stroke and smoking

The multivariate regression analysis showed that BSH increased by 3.99-times (odds ratio [OR] 3.99, 95% CI 2.05–7.78, *p* < 0.01) when RWT was > 0.42, but not when LVMI increased (OR 0.16, 95% CI 0.02–1.19, *p* = 0.07). Detailed information is presented in Table 3. The results of the subgroup analysis showed that there were no interactions between BSH and the covariates of age, BMI, sex, diabetes mellitus, CHD, stroke, and smoking in relation to the increase in RWT. The details are shown in Fig. 1.

**Table 3:** Multivariable logistic regression analysis of BSH for LVMI and RWT increasing

	Increased LVMI^c^				RWT > 0.42			
	Crude OR (95%CI)	*p*	Adjusted OR (95%CI)^a^	*p*	Crude OR (95%CI)	*p*	Adjusted OR (95%CI)^b^	*p*
BSH	1.16 (0.02, 1.18)	0.07	0.16 (0.02, 1.19)	0.07	5.03 (2.68, 9.44)	< 0.01	3.99 (2.05, 7.78)	< 0.01

*BSH* Basal septal hypertrophy; *LVMI* Left ventricular mass index; *RWT* Relative wall thickness

^a^Adjusted for age, sex, obesity, hypertension, diabetes mellitus and heart rate

^b^Adjusted for age, sex, obesity, hypertension, diabetes mellitus, heart rate and LVMI

^c^Increased LVMI was defined as LVMI o > 115 g/m^2^ (male)or > 95 g/m^2^ (female)

## Discussion

BSH detected by routine echocardiography is prominent in older individuals. In this study, BSH was independently associated with an increase in RWT, but not with an increase in LVMI. The subgroup analysis showed no interactions between BSH and the covariates of age, sex, BMI, diabetes mellitus, CHD, stroke, and smoking status in relation to the increase in RWT.

The mechanism leading to BSH is unclear. BSH is recognized early in patients with essential hypertension [9]. This localized thickening decreases in response to antihypertensive treatment [10]. Thus, BSH might be valuable to detect masked hypertension in the general population [3]. Central blood pressure correlates with basal IVS thickness, but not with mid-IVS thickness [11]. Thus, stricter blood pressure management and hypertension screening should be conducted in patients with BSH. A previously published cohort study showed that hypertensive subjects with BSH were older than non-BSH subjects and had a higher BMI and systolic blood pressure [12]. In our study, patients in the BSH group were older than those in the non-BSH group, while the systolic blood pressure tended to be higher in patients with BSH than in those without, although the difference was not significant. There was no difference in the prevalence of hypertension between the BSH group and the non-BSH group, which may be because the overall population was older and the prevalence of hypertension was higher in our study. In the present study, the BSH group had a higher incidence of diabetes mellitus, which is in contrast to the study of Loncaric et al., who observed no difference in the incidence of diabetes mellitus between the BSH group and the non-BSH group [12], which might be due to the younger age and lower prevalence of diabetes in this group.

A previous cohort study showed that hypertensive patients with BSH had a higher LV ejection fraction and lower LV end-diastolic and end-systolic volumes, while no significant differences were observed in left atrial size between the two groups [12]. In the present study, we found that LVEDD was lower in patients with BSH (*p* < 0.05). Unlike previous studies [12], the LVMI in the BSH group was lower than in the non-BSH group in our study, which may be related to the baseline level of the included populations. Early clinical studies suggested that BSH is related to LV diastolic function [2]. Another study showed that BSH is less likely to cause increased LV stiffness without LV hypertrophy [13], but that it can affect LV diastolic function during stress [14]. BSH is related to cardiac function in patients with hypertension with well-controlled blood pressure. Basal and mid-posterior wall systolic deformation, LV diastolic function, and left atrial function are decreased in these patients [12]. Thus, such patients are prone to heart failure with preserved ejection fraction.

In the present study, concentric remodeling was the most frequent LV geometry since the higher prevalence of hypertension in our cohort. CR was the most frequent LV geometry in patients with BSH as well which imply that CR was the main LV geometry type in BSH people. A retrospective analysis of a large population (n = 35,602) showed an abnormal LV geometry in 46% of patients, with concentric remodeling present in 35% of patients and LV hypertrophy present in 11% of patients [5]. Patients with hypertension had race-related differences in LV geometry and RWT. A descriptive study previously reported that Africans exhibited a greater IVS thickness and RWT than Caucasians [15]. A study with a mean follow-up period of 2.5 years assessed the effect of potential changes in cardiac structure and found that 1610 patients (45%) demonstrated no change in LV geometry and maintained a pattern of concentric remodeling, 439 patients (12%) progressed to LV hypertrophy, and 1567 patients (43%) converted to a normal LV geometry. There was a strong relationship between an abnormal LV geometry and all-cause mortality. Patients with concentric remodeling and LV hypertrophy exhibited considerably higher mortality than patients with a normal LV geometry [5]. An American population-based case–control study showed that concentric remodeling is associated with stroke risk [16]. A prospective study showed that all-cause mortality was significantly more likely in patients with concentric remodeling (hazard ratio 1.417, 95% CI 1.045–1.920) [17]. Therefore, follow-up and risk factor control of patients with concentric remodeling should be strengthened to reduce the occurrence of cardiovascular events, but we did not pay sufficient attention to LV geometry in a real-world setting.

In this study, BSH independently correlated with an increase in RWT. A previous study showed that an increase in RWT is a strong independent predictor of mortality [5]. RWT significantly increases stroke risk, but no interactions have been detected between RWT and LVM [16]. A prospective study showed that RWT is an independent predictor of all-cause and cardiovascular mortality in patients who experience ischemic stroke, whereas the association between LVMI and all-cause death is not significant [17]. In the present study, hypertensive patients with BSH demonstrated a greater RWT and accounted for the highest proportion of patients with concentric remodeling. Therefore, if LV geometry is routinely measured in clinical practice, cardiovascular risk in patients with BSH may be increased.

Our study has several limitations that should be noted. First, because of the cross-sectional study design, a causal relationship between BSH and LV geometry could not be determined. Prospective studies examining whether BSH is predictive of LV geometry and cardiovascular events are required. Second, the majority of patients were aged > 40 years; thus, our findings may not reflect the characteristics of BSH in a younger population. Finally, according to our inclusion criteria, some patients with hypertrophic cardiomyopathy may have been included in this study. And all patients were volunteer, which might affect the result in general population.

## Conclusion

In this study, we showed that patients with BSH accounted for the highest proportion of patients with concentric remodeling. BSH independently correlated with an increase in RWT. Subgroup analysis showed that there were no interactions between BSH and the covariates of in relation to the increase in RWT.

## Data Availability

The datasets generated and analyzed during the current study are not publicly available owing to data security issues, but are available from the corresponding author on reasonable request.

## Abbreviations

BSH: Basal septal hypertrophy
BMI: Body mass index
CHD: Coronary heart disease
CI: Confidence interval
CR: Concentric remodeling
CH: Concentric hypertrophy
EH: Eccentric hypertrophy
IVS: Interventricular septal
LV: Left ventricular
LVM: Left ventricular mass
LVMI: Left ventricular mass index
LVEDD: Left ventricular end-diastolic dimension
LVESD: Left ventricular end-systolic dimension
NG: Normal geometry
OR: Odds ratio
PWT: Posterior wall thickness
PWTd: Posterior wall thicknesses at LV end-diastole
RWT: Relative wall thickness
SWTd: Septal wall thicknesses at LV end-diastole
